# Outcomes of renin‐angiotensin inhibitors following transcatheter aortic valve implantation

**DOI:** 10.1002/clc.24187

**Published:** 2023-11-07

**Authors:** Alireza Hosseinpour, Rahul Gupta

**Affiliations:** ^1^ School of Medicine Shiraz University of Medical Sciences Shiraz Iran; ^2^ Lehigh Valley Heart Institute Lehigh Valley Health Network Allentown Pennsylvania USA

(1) This paper is not under consideration elsewhere; (2) None of the paper's contents have been previously published (3) All authors have read and approved the manuscript; and (4) All authors take responsibility for all aspects of the reliability and freedom from bias of the data presented and their discussed interpretation.

The mechanical overload as a consequence of aortic stenosis (AS) has been supposedly associated with activation of cardiac renin‐angiotensin system (RAS). This activation leads to upregulation of angiotensin‐converting enzyme (ACE) messenger RNA and hence, myocardial ACE. This process contributes to increased myocardial collagen and fibrosis leading to cardiac failure.^1^ Recently, several clinical studies have investigated the potential beneficiary effects of RAS inhibitors (ACE inhibitors and angiotensin receptor blockers [ARBs]) in patients with AS undergoing transcatheter aortic valve implantation (TAVI). Studies have shown variables effects of RAS inhibitors on clinical outcomes in patients undergoing TAVI.^2^, ^3^ We sought to provide an updated data regarding the potential effects of RAS inhibitors in AS patients after TAVI by performing a meta‐analysis of clinical outcomes.

A literature search was conducted through digital databases in a systematic approach to find all the articles providing data on the outcomes of TAVI patients compared between patients taking RAS inhibitors and the ones not taking this medication. Reference sections of eligible articles and similar meta‐analyses were screened for additional records. Primary endpoints were all‐cause mortality, cardiac mortality, and rehospitalization at the longest available follow‐up. Long‐term myocardial infarction (MI), stroke and new pacemaker implantation and acute kidney injury (AKI) were listed as secondary outcomes of interest. Qualitative assessment of the studies were performed using Newcastle‐Ottawa Quality Assessment Form for Cohort Studies. Absolute number of events and total population were extracted from each group and relative risk (RR) and 95% confidence interval (CI) were calculated in a random‐effects analysis using “meta” package in RStudio software version 1.3.959. For all‐cause mortality, in addition to measurement of RR, hazard ratio (HR) and 95% CI were retrieved and log HR and standard error were calculated to combine all the available HRs and pool an overall HR of all‐cause mortality using inverse variance method. A pooled value with 95% CI not crossing the line of one was considered a statistically significant result.

Following assessment of the retrieved records, nine articles^2^, ^3^, ^4^, ^5^, ^6^, ^7^, ^8^, ^9^, ^10^ with 36 322 participants (17 730 treated with RAS inhibitors and 18 592 without RAS inhibitors) were included for quantitative synthesis (Table 1). In terms of quality assessment, the majority of the included studies had good quality (Supporting Information S1: Table 1). For all‐cause mortality, all of the individual studies showed a decreased risk in the group taking RAS inhibitors except one.^2^ For cardiac mortality, all of the included studies showed a significant risk reduction in the RAS inhibitor group. Treatment with RAS inhibitors was significantly associated with lower all‐cause mortality (RR 0.73, 95% CI 0.62−0.85, *p* = .0016), cardiac‐related mortality (RR 0.66, 95% CI 0.57−0.76, *p* = .0029), and rehospitalization rates (RR 0.83, 95% CI 0.77−0.90, *p* = .0024). Pooled analysis showed that patients taking a RAS inhibitor following TAVI had significantly reduced all‐cause mortality HR compared to the patients not on it (pooled HR (95% CI) = 0.70 (0.60−0.81), *p* < .0001). RAS inhibitor therapy was not associated with a different risk compared to patients not taking a RAS inhibitor in terms of stroke (RR 0.44, 95% CI 0.08−2.39) and MI (RR 0.95, 95% CI 0.15−5.93). Short‐term postprocedural outcomes including AKI (RR 1.04, 95% CI 0.72−1.49) and new pacemaker implantation (RR 1.09, 95% CI 0.55−2.17) were also not different among the two groups (Figure 1).

**Table 1:** Baseline characteristics of the included studies.

Study	Study design	No. of patients		Age		Male gender (%)		LVEF (%)		Hypertension (%)		Diabetes (%)		Dyslipidemia (%)		CAD (%)		Chronic renal disease (%)	
		RASI	No RASI	RASI	No RASI	RASI	No RASI	RASI	No RASI	RASI	No RASI	RASI	No RASI	RASI	No RASI	RASI	No RASI	RASI	No RASI
Chen 2020	Analysis cohort of PARTNER 2 A&B randomized cohorts + PARTNER 2 S3 registries	1736	2243	81.7 ± 7.2	82.9 ± 7.7	60.8	58.4	54.5 ± 13.7	54.9 ± 13.3	96.3	89.7	40.6	32.5	84.4	79.4	81.0	76.0	7.2	9.8
Cubeddu 2023	Retrospective analysis of Optum® Clinformatics® Data Mart Database	3172	5840	79.3 ± 7.2	80.4 ± 6.7	51.9	54.4	ND	ND	97.5	92.8	50.6	41.8	ND	ND	86.8	86.4	34.8	36.9
**Fischer‐**Rasokat 2022	Retrospective analysis of a single‐center observational registry	2227	635	82 (78.7‐85.0)	82 (78.2‐85.6)	47.8	47.1	65 (55‐65)	65 (55‐65)	93.4	82	32.9	32.6	ND	ND	60.9	53.4	ND	ND
Inohara 2018	Retrospective cohort study	8468	12844	82.3 ± 6.8	82.9 ± 6.9	53.0	52.6	51.1 ± 12.1	52.6 ± 10.8	93.9	87.9	39.8	34.1	ND	ND	ND	ND	45.3	52.7
Kaewkes 2020	Retrospective analysis of Cedars‐Sinai Medical Center database	349	415	81.4 ± 7.7	82.9 ± 8.7	57	60	59.3 ± 14.4	58.7 ± 23.7	94	86	34	24	ND	ND	43	39	75	73
Klinkhammer 2019	Retrospective cohort study	71	98	77.8 ± 7.9	80.1 ± 7.5	40	60	55.6 ± 14.1	58.6 ± 11.6	68	75	36	31	67	83	53	75	33	56
Ledwoch 2021	Prospective observational study	225	98	80.7 ± 6.6	79.8 ± 9.3	56	54	52.4 ± 10.1	51.7 ± 11.2	94.2	81	25.8	19	65	52	76	60	63	54
Ochiai 2017	Prospective cohort of OCEAN‐TAVI registry	371	189	84.2 ± 5.0	54.8 ± 5.0	29.9	24.3	62.9 ± 13.1	63.3 ± 11.9	83.8	61.4	27.8	24.3	ND	ND	41.5	33.9	66.3	57.1
Rodriguez‐Gabella 2019	Multicenter retrospective cohort of RASTAVI registry	1622	1163	80.8 ± 7.0	80.7 ± 7.2	45.1	46.9	57.4 ± 13.9	58.9 ± 13.4	85.6	74.8	36.4	31.7	57	52.2	39.1	32.9	37.3	42.5

Abbreviations: CAD, coronary artery disease; LVEF, left ventricle ejection fraction; ND, no data; RASI, renin‐angiotensin inhibitor.

**Figure 1:**
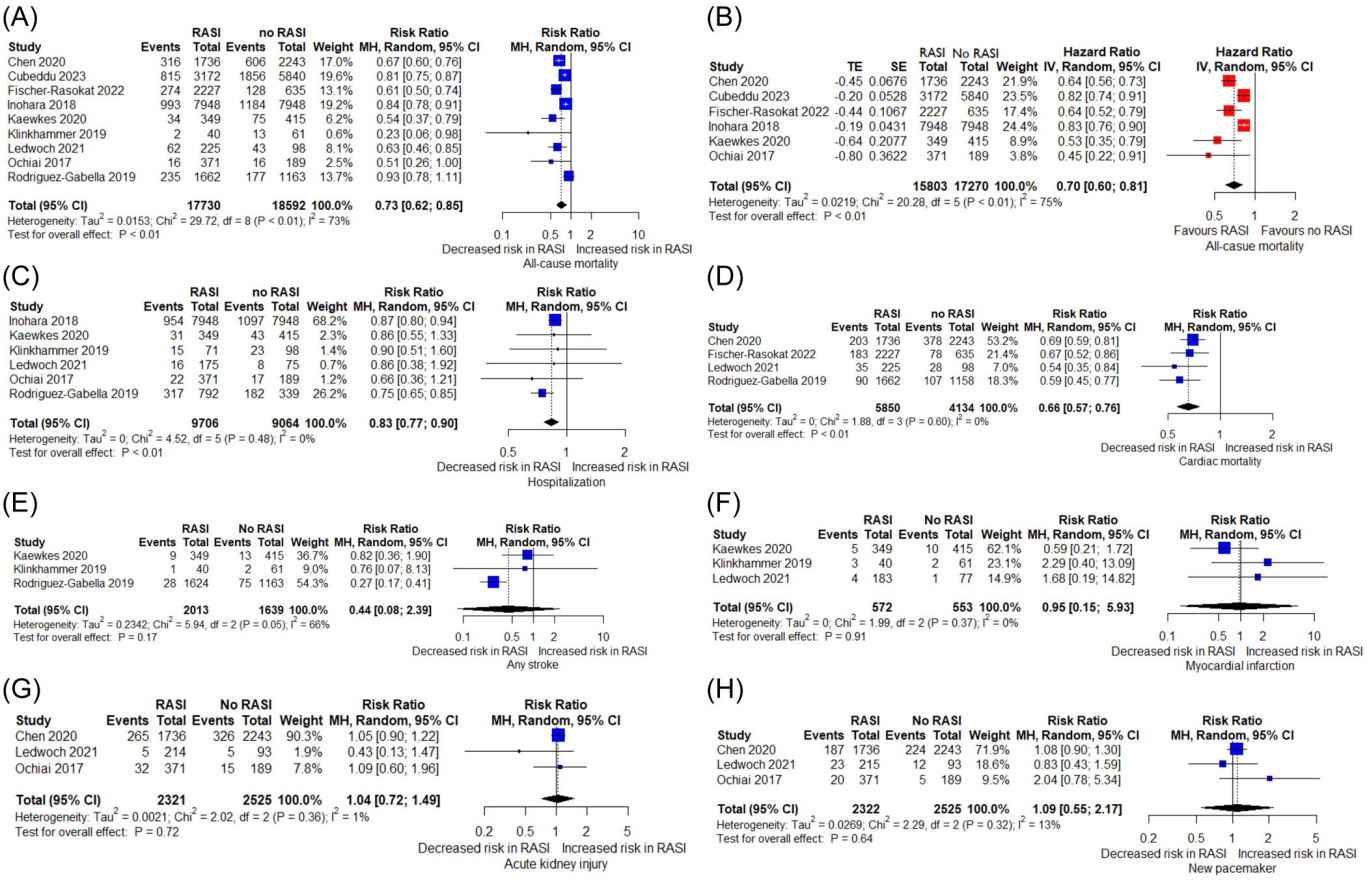
The pooled effect of RAS inhibitors vs no RAS inhibitors in patients with AS following TAVI on A, all‐cause mortality (relative risk); B, all‐cause mortality (hazard ratio); C, rehospitalization; D, cardiac‐related mortality; E, stroke; F, myocardial infarction; G, acute kidney injury; H, new pacemaker implantation.

Our study showed that RAS inhibitor therapy following TAVI is associated with long‐term survival benefit and lower rates of rehospitalization. Better survival was derived from both all‐cause and cardiac mortality. A trend was observed towards decreased risk of all‐cause and cardiovascular mortality and hospitalization rate in the majority of the included studies. It is noteworthy that for better interpretation of the results, it is of great importance to check the raw data from each of the individual studies as differences in the studies may rise from different study design, study population, intervention, outcomes, and potential covariates. Contrary to these results, no significant impact was observed on stroke and MI rates in patients taking RAS inhibitor medications. Also, RAS inhibitors did not appear to have an impact on short‐term outcomes (AKI and pacemaker implantation), although available data on short‐term outcomes were limited. The findings of the present meta‐analysis support the beneficial effects of RAS inhibitors (ACE inhibitors/ARBs) on mortality and rehospitalization, although short‐term outcomes may not be influenced by prescription of RAS inhibitors post‐TAVI. The lower mortality rate in patients taking a RAS inhibitor can be attributed to a decline in left ventricular global pressure overload and hence, prevention of hypertrophy and irreversible ventricular damage.^4^ It has been shown that patients who continue to use RAS inhibitors at long‐term show a greater survival benefit than those who do not adhere to the medication.^5^ On top of that, this treatment effect is significant regardless of baseline left ventricular ejection fraction, age, and gender^4^, ^5^ although other studies have showed significant benefit only in patients older than 80 years old^4^ and male gender.^6^ An interesting finding in subgroup analysis of the studies by Cubeddu and Fischer‐Rasokat et al.^5^, ^6^ showed that concomitant use of RAS inhibitors with beta‐blockers was significantly associated with lower all‐cause mortality.

In a recent meta‐analysis of seven studies on outcomes of RAS inhibitors following TAVI, the authors showed that prescription of RAS inhibitors after TAVI resulted in lower all‐cause and cardiovascular mortality and rehospitalization for heart failure.^11^ In our study, which is the most updated meta‐analysis by adding two recent studies,^5^, ^9^ we compared additional outcomes including stroke and myocardial infarction rate, and also two postprocedural parameters (AKI and new pacemaker implantation). In our analysis, we also combined all the reported HRs and calculated an overall hazard for all‐cause mortality.

There are some limitations in this meta‐analysis that should be considered. First, the included studies were among non‐randomized retrospective cohorts or post hoc analyses. This can cause a significant selection bias and may have influenced the results. As in every nonrandomized study, presence of baseline confounders may account for significant heterogeneity in our results. This issue was more pronounced in the studies not adjusting the baseline parameters. Data were not categorized clearly in the studies to see if patients on RAS inhibitors were already on the medication before TAVI as the beneficial effect of RAS inhibitors may be due to the baseline treatment. Studies reporting stroke, myocardial infarction, and other postprocedural outcomes were limited. Proper subgroup analyses could not be performed to assess the effect of co‐prescription of RAS inhibitors and beta‐blockers since data reported in the studies were not classified.

## CONCLUSION

In this study, we showed that TAVI patients taking RAS inhibitors had long‐term survival benefit with lower rates of rehospitalization compared with the patients not taking a RAS inhibitor. However, this effect was not displayed on short‐term outcomes and stroke rate. Future meta‐analyses with individual participant data are warranted to provide data on clinical outcomes in different subgroups including co‐prescription with beta‐blockers and other baseline characteristics to optimize selection of the patients that may benefit more from RAS inhibitor therapy.

## AUTHOR CONTRIBUTIONS

*Conception and design*: Alireza Hosseinpour and Rahul Gupta. *Drafting of the manuscript and revising it critically for important intellectual content*: Alireza Hosseinpour and Rahul Gupta. *Final approval of the manuscript submitted*: Rahul Gupta.

## CONFLICT OF INTEREST STATEMENT

The authors declare no conflict of interest.

## Supporting information

Supporting information.

## Data Availability

The data underlying this article will be shared on reasonable request from the corresponding author.

## References

[1] Fielitz J , Hein S , Mitrovic V , et al. Activation of the cardiac renin‐angiotensin system and increased myocardial collagen expression in human aortic valve disease. JACC. 2001;37(5):1443‐1449. 11300459 10.1016/s0735-1097(01)01170-6

[2] Rodriguez‐Gabella T , Catalá P , Muñoz‐García AJ , et al. Renin‐Angiotensin system inhibition following transcatheter aortic valve replacement. JACC. 2019;74(5):631‐641. 31370954 10.1016/j.jacc.2019.05.055

[3] Ledwoch J , Olbrich I , Poch F , et al. Dose‐Dependent effect of Renin‐Angiotensin system blockade following transcatheter aortic valve replacement. Can J Cardiol. 2021;37(3):443‐449. 32835854 10.1016/j.cjca.2020.08.014

[4] Chen S , Redfors B , Nazif T , et al. Impact of renin‐angiotensin system inhibitors on clinical outcomes in patients with severe aortic stenosis undergoing transcatheter aortic valve replacement: an analysis of from the PARTNER 2 trial and registries. Eur Heart J. 2020;41(8):943‐954. 31711153 10.1093/eurheartj/ehz769 PMC8204653

[5] Cubeddu RJ , Murphy SME , Asher CR , et al. Association of ACEI/ARB and statin prescribing patterns with mortality after transcatheter aortic valve replacement (TAVR): findings from real‐world claims data. Am Heart J. 2023;258:27‐37. 36596333 10.1016/j.ahj.2022.12.012

[6] Fischer‐Rasokat U , Bänsch C , Renker M , et al. Effects of renin‐angiotensin system inhibitor type and dosage on survival after transcatheter aortic valve implantation. Euro Heart J Cardiovasc Pharmacother. 2022;8(8):815‐824. 10.1093/ehjcvp/pvac027 35441662

[7] Inohara T , Manandhar P , Kosinski AS , et al. Association of renin‐angiotensin inhibitor treatment with mortality and heart failure readmission in patients with transcatheter aortic valve replacement. JAMA. 2018;320(21):2231‐2241. 30512100 10.1001/jama.2018.18077 PMC6583475

[8] Kaewkes D , Ochiai T , Flint N , et al. Optimal medical therapy following transcatheter aortic valve implantation. Am J Cardiol. 2021;141:62‐71. 33221263 10.1016/j.amjcard.2020.11.010

[9] Klinkhammer B . Renin‐angiotensin system blockade after transcatheter aortic valve replacement (TAVR) improves intermediate survival. J Cardiovasc Thorac Res. 2019;11(3):176‐181. 31579456 10.15171/jcvtr.2019.30 PMC6759619

[10] Ochiai T , Saito S , Yamanaka F , et al. Renin‐angiotensin system blockade therapy after transcatheter aortic valve implantation. Heart. 2018;104(8):644‐651. 28986405 10.1136/heartjnl-2017-311738

[11] Wang S , Lin X , Guan Y , Huang J . The clinical outcomes of reni‐angiotensin system inhibitors for patients after transcatheter aortic valve replacement: A systematic review and meta‐analysis. Front Cardiovasc Med. 2022;9:963731. 36035924 10.3389/fcvm.2022.963731 PMC9402980

