# Supplementary material for: Outcomes of renin‐angiotensin inhibitors following transcatheter aortic valve implantation
Source: Clin Cardiol. 2023 Nov 7;47(2):e24187. doi: 10.1002/clc.24187 (PMC10826235; doi:10.1002/clc.24187)
Supplement: Supplementary file 1 — Supporting information. [file CLC-47-e24187-s001.docx]

**Table S1:** Newcastle-Ottawa Quality Assessment Form for Cohort Studies

| **Study** | **Selection** | | | | **Comparability** (analysis controlled for confounders) | **Outcome** | | | **Overall assessment** |
| --- | --- | --- | --- | --- | --- | --- | --- | --- | --- |
|  | Representativeness | Selection of the non-exposed | Ascertainment of exposure | Demonstration of non-presence of outcome at the start |  | Assessment | Duration of follow-up | Adequacy of follow-up |  |
| **Chen 2020 (1)** | * | * | * | * | * | * | * | * | Good |
| **Cubeddu 2023 (2)** | * | * | * | * | * | * | * | * | Good |
| **Fischer-Rasokat 2022 (3)** |  | * | * | * | * | * | * | * | Good |
| **Inohara 2018 (4)** | * | * | * | * | ** | * | * | * | Good |
| **Kaewkes 2020 (5)** |  | * | * | * |  | * | * |  | Poor |
| **Klinkhammer 2019 (6)** |  | * | * | * |  | * | * |  | Poor |
| **Ledwoch 2021 (7)** |  | * | * | * | * | * | * | * | Good |
| **Ochiai 2017 (8)** | * | * | * | * | * | * | * | * | Good |
| **Rodriguez-Gabella 2019 (9)** | * | * | * | * | * | * | * | * | Good |
